# Bat Lyssaviruses, Northern Vietnam

**DOI:** 10.3201/eid2001.130813

**Published:** 2014-01

**Authors:** Anh Thi Kieu Nguyen, Thu Tuyet Nguyen, Akira Noguchi, Dong Vinh Nguyen, Giang C. Ngo, Vu Dinh Thong, Babatunde Olowokure, Satoshi Inoue

**Affiliations:** National Institute of Hygiene and Epidemiology, Hanoi, Vietnam (A.T.K. Nguyen, T.T. Nguyen, D.V. Nguyen, G.C. Ngo);; National Institute of Infectious Diseases, Tokyo, Japan (A. Noguchi, S. Inoue);; Institute of Ecology and Biological Resources, Hanoi (V.D. Thong);; World Health Organization Vietnam Country Office, Hanoi (B. Olowokure)

**Keywords:** bats, rabies, lyssaviruses, viruses, serology, Vietnam

**To the Editor:** Bats have been associated with a wide diversity of viruses, including lyssaviruses, which can cause rabies. Currently, 12 distinct species of lyssaviruses have been classified worldwide; 3 of these were isolated from bats in northern and central Asia (*1*). In addition, 3 putative novel bat lyssaviruses (Boklob, Ikoma, and Leida) have recently been described and are awaiting taxonomic assessment (*1*,*2*). Surveys for lyssaviruses in bat reservoirs in several countries in Southeast Asia, such as the Philippines, Cambodia, and Thailand, showed that bat lyssaviruses are naturally circulating in insectivorous and frugivorous bats (*3*–*5*).

Rabies is endemic to Vietnam, and ≈100 human deaths caused by rabies are reported annually; most are attributable to canine rabies (*6*). Although bat-associated rabies cases have not been reported in humans or animals in Vietnam, this finding might be caused by lack of a suitable reporting system. The limited understanding of the extent of lyssavirus circulation in Vietnam and its potential effect on public and animal health prompted this surveillance study.

This study was approved by the ethics committee of The National Institute of Hygiene and Epidemiology, and all capture and experimental procedures complied with institute guidelines for bat capture and use. During May–September 2011, a total of 926 bats were collected from 6 northern provinces in Vietnam (Technical Appendix Figure). Bats were classified by using a gross morphology key (*7*). Blood and brain samples were obtained after anesthetizing bats by intramuscular injection with 0.05–0.1 mg ketamine hydrochloride.

All bat brains were tested for lyssavirus by using reverse transcription PCR (*8*) and for lyssavirus antigens by using a direct fluorescence antibody test (*9*) using fluorescein isothiocyanate–conjugated monoclonal antibodies (Fujirebio Diagnostic, Inc, Malvern, PA, USA). The mouse inoculation test was conducted by using brains of 13 bats that died during capture and transport (*9*).

A total of 789 bat serum samples were of sufficient quality and quantity to be screened for neutralizing antibodies against rabies virus (RABV) strain CVS-11, European bat lyssavirus-1 (EBLV-1), and Duvenhage virus (DUVV). We also tested for neutralizing antibodies against Lagos bat virus (LBV) and Mokola virus in 535 samples by using a modified rapid fluorescent focus inhibition test (*10*). A sample was defined as positive for neutralizing antibodies if at a serum dilution of 1:10 a ≥90% reduction was observed in the number of infectious fields in comparison with the virus control.

All 926 bats collected were identified to 25 species. Of these species, 23 were Microchiropteran species and 2 were Megachiropteran species (Table). None of the 926 bat brain samples showed evidence of lyssavirus antigens or virus RNA by direct fluorescence antibody test and reverse transcription PCR, respectively. No virus was isolated by the mouse inoculation test.

**Table:** Screening of bat serum samples for neutralizing antibodies against lyssaviruses, northern Vietnam*

Bat species (no.captured)	Virus strains				Total, no. positive/no. tested†
	RABV	DUVV	EBLV-1	LBV	
Microchiropteran				0	NA
*Aselliscus stoliczkanus* (45)	0	0	1	0	1/29
*Hipposideros alongensis sungi* (19)	0	3	2	0	5/16
*Hipposideros armiger* (11)	0	0	3	0	3/9
*Hipposideros larvatus* (138)	26	55	53	0	63/126
*Hypsugo* sp. 1 (16)	0	0	3	0	3/12
*Hypsugo* sp. 2 (17)	0	7	2	0	7/15
*Ia io* (46)	4	1	6	0	8/44
Miniopterus cf. fuliginosus (27)	0	0	2	0	2/23
*Miniopterus* sp. (13)	0	0	0	0	0/10
*Myotis* sp. 1 (13)	1	0	2	0	3/8
*Myotis* sp. 2 (11)	0	0	0	0	0/9
*Pipistrellus* sp. (19)	0	0	0	0	0/10
*Rhinolophus affinis* (11)	0	0	0	0	0/7
*Rhinolophus cf. microglobosus* (40)	0	1	2	0	2/34
*Rhinolophus* cf. *pearsonii* (21)	0	0	0	0	0/16
*Rhinolophus* cf. *pusillus* (9)	0	0	0	0	0/6
*Rhinolophus macrotis* (large) (18)	0	0	0	0	0/14
*Rhinolophus macrotis* (small) (16)	0	3	1	0	3/6
*Rhinolophus pusillus* (24)	0	1	2	0	2/19
*Tadarida plicata* (65)	17	0	9	1	19/55
*Taphozous* cf. *melanopogon* (223)	9	0	22	0	22/203
*Taphozous* sp. (25)	0	0	1	0	1/19
*Taphozous theobaldi* (74)	30	0	31	3	45/74
Megachiropteran					NA
*Eonycteris spelaea* (9)	3	0	1	0	4/9
*Rousettus* sp. (16)	0	0	0	0	0/16
Total	90	71	142	4	193/789
No. positive/no. tested (%)	90/789 (11.4)	71/789 (9)	142/789 (18)	4/535 (0.75)	193/789 (24.5)

*RABV, rabies virus CVS 11; DUVV, Duvenhage virus; EBLV-1, European bat lyssavirus-1; LBV, Lagos bat virus; NA, not applicable. †Values in rows may be higher than those in the total because of reactivity of individual serum samples against >1 lyssavirus.

Of the 789 bat serum samples tested, 193 (24.5%) were positive for neutralizing antibodies against lyssaviruses. Ninety (11.4%) of 789 bat serum samples had neutralizing antibodies against RABV, 71 (9.0%) against DUVV, 142 (18.0%) against EBLV-1, and 4 (0.75%) against LBV. No bat serum was positive for neutralizing antibodies against Mokola virus. Neutralizing antibodies against the 5 lyssavirus genotypes tested were found in 16 Microchiropteran and 1 Megachiropteran species (Table).

Of the 193 serum samples positive for neutralizing antibodies against lyssaviruses, 65 (33.7%) also neutralized ≥1 of the remaining viruses tested. Twenty-five samples that were positive for RABV were negative for the other viruses; 103 samples were negative for RABV but positive for ≥1 of EBLV-1, DUVV, and LBV. Different titers of neutralizing antibodies against different lyssaviruses were found in some bats (Technical Appendix Table).

This study provides serologic evidence of lyssavirus-neutralizing antibodies in bats in northern Vietnam. Because no virus was isolated, we could not conclude to which virus or viruses these bats had been exposed. Positive results for antibodies to multiple lyssaviruses, including RABV, found in some bats might have been caused by cross-neutralization of other viruses. The absence of consistent reactivity patterns suggests exposure of these bats to the tested lyssaviruses or another unknown lyssavirus. These findings are similar to findings reported from other parts of Asia (*3*–*5*).

Information on lyssavirus circulation in bat populations in Vietnam should be made available to public health authorities, clinicians, and the general public to increase awareness of the risk for rabies transmission from bats; improve recognition, documentation, and reporting of bat exposure to rabies surveillance systems; and increase consideration of the need for post exposure prophylaxis after receiving a bat bite. Our data suggest that several lyssaviruses are circulating among bats in northern Vietnam, and a substantial proportion have neutralizing antibodies to RABV. Further investigations are required, particularly of sick and dying bats, to determine the implications of these findings for human health.

Technical Appendix Neutralization antibody titers against lyssaviruses in bats, by bat species and location, and bat collection sites and serum samples tested for lyssavirus, northern Vietnam.
