# Supplementary material for: Bat Lyssaviruses, Northern Vietnam
Source: Emerg Infect Dis. 2014 Jan;20(1):161–3. doi: 10.3201/eid2001.130813 (PMC3884725; doi:10.3201/eid2001.130813)
Supplement: Technical Appendix — Neutralization antibody titers against lyssaviruses in bats, by bat species and location, and bat collection sites and serum samples tested for lyssavirus, northern Vietnam. [file 13-0813-Techapp-s1.pdf]

Article DOI: http://dx.doi.org/10.3201/eid2001.130813

Bat Lyssaviruses, Northern Vietnam

Technical Appendix

Technical Appendix Table. Neutralization antibody titers against lyssaviruses in bats, by bat species and location, northern Vietnam*

		Neutralizing antibody titer†			
Location	Bat species	RABV	DUVV	EBLV-1	LBV
Tuyen Quang	*Eonycteris spelaea*	20	0	0	0
	Eonycteris spelaea	0	0	20	0
	Ia Io	20	0	0	0
	Ia Io	0	20	0	0
	Ia Io	0	20	0	0
	Ia Io	10	0	20	0
	Ia Io	10	10	40	0
Lang Son	*Taphozous* cf. *melanopogon*	20	0	10	0
	Taphozous cf. *melanopogon*	10	0	20	0
	Taphozous cf. *melanopogon*	10	0	20	0
	Taphozous cf. *melanopogon*	0	0	20	0
	Taphozous cf. *melanopogon*	10	0	20	0
	Tadarida plicata	20	0	40	0
	Tadarida plicata	0	0	20	0
Bac Giang	*Taphozous theobaldi*	10	0	20	10
	Taphozous theobaldi	20	0	40	0
	Taphozous theobaldi	0	0	20	10
	Taphozous theobaldi	20	0	0	0
	Taphozous theobaldi	0	0	0	10
	Taphozous theobaldi	20	0	40	0
	Taphozous theobaldi	0	0	20	0
	Taphozous theobaldi	0	0	0	10
	Tadarida plicata	20	0	10	0
Phu Tho	*Hipposideros larratus*	0	0	0	0
	Hipposideros larratus	20	10	40	0
	Hipposideros larratus	10	20	0	0
	Rhinolophus macrotis (small)	0	0	20	0
Hoa Binh	*Taphozous* cf. *melanopogon*	20	0	40	0
	Taphozous cf. *melanopogon*	0	0	20	0

*RABV, rabies virus; DUUV, Duvenhage virus; EVLV-1, European bat lyssavirus-1; LBV, Lagos bat virus.
†Titer of neutralizing antibodies was calculated as the initial serum volume plus an equal volume of challenge virus that showed ≥90% reduction in number of infectious fields compared with virus control.

Place of bat collection	Bat serum samples positive for neutralizing antibodies				
	RABV	DUVV	EBLV-1	LBV	MOKV
Tuyen Quang	+	+	+	-	-
Yen Bai	+	+	+	-	-
Lang Son	+	-	+	-	-
Bac Giang	+	-	+	+	-
Phu Tho	+	+	+	-	-
Hoa Binh	+	-	+	-	-

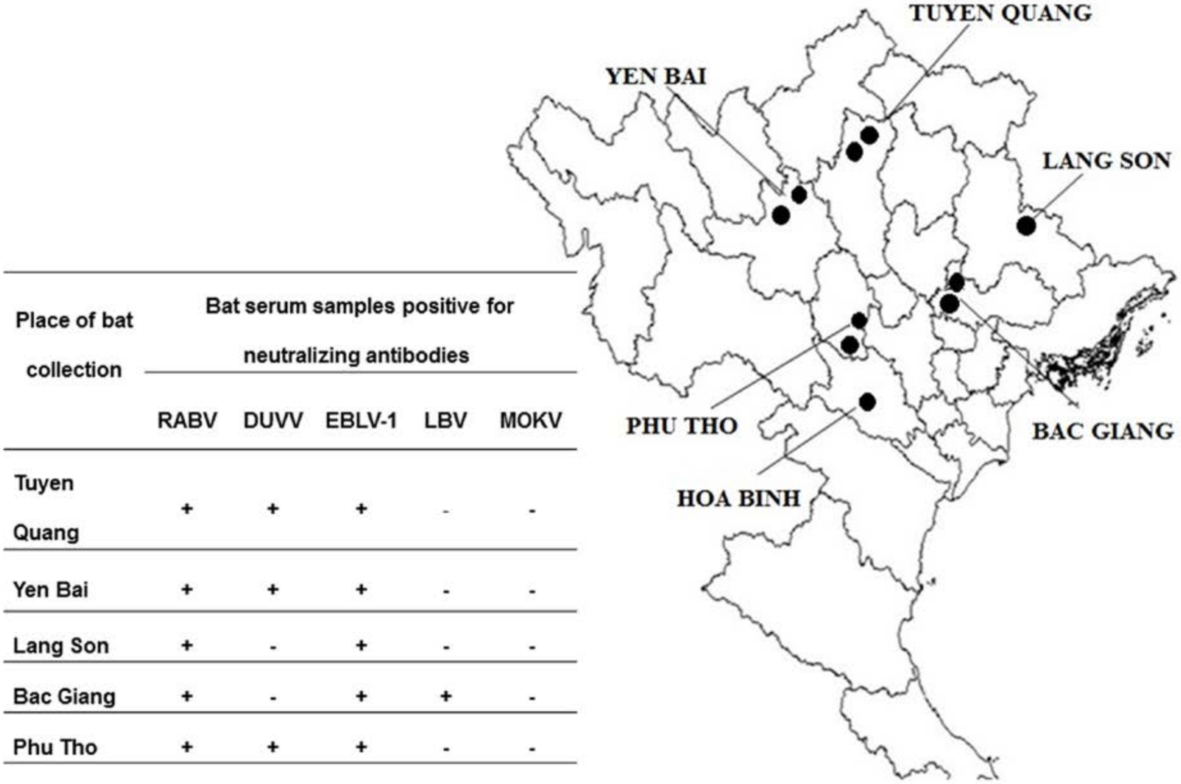

Technical Appendix Figure. Bat collection sites and serum samples tested for lyssavirus, northern Vietnam. RABV, rabies virus; DUUV, Duvenhage virus; EBLV-1, European bat lyssavirus-1; LBV, Lagos virus; MOKV, Mokola virus; +, positive; –, negative.
